# Duration of Ice Hockey Play and Chronic Traumatic Encephalopathy

**DOI:** 10.1001/jamanetworkopen.2024.49106

**Published:** 2024-12-04

**Authors:** Bobak Abdolmohammadi, Fatima Tuz-Zahra, Madeline Uretsky, Raymond Nicks, Sydney Mosaheb, Jacob Labonte, Eukyung Yhang, Shruti Durape, Brett Martin, Joseph Palmisano, Christopher Nowinski, Jonathan D. Cherry, Victor E. Alvarez, Bertrand R. Huber, Kristen Dams-O’Connor, John Crary, Brigid Dwyer, Daniel H. Daneshvar, Lee E. Goldstein, Rhoda Au, Douglas I. Katz, Neil W. Kowall, Robert C. Cantu, Robert A. Stern, Michael L. Alosco, Thor D. Stein, Yorghos Tripodis, Ann C. McKee, Jesse Mez

**Affiliations:** 1 Boston University Alzheimer’s Disease Research Center, Boston University Chobanian and Avedisian School of Medicine, Boston, Massachusetts; 2 Boston University CTE Center, Boston University Chobanian and Avedisian School of Medicine, Boston, Massachusetts; 3 Department of Biostatistics, Boston University School of Public Health, Boston, Massachusetts; 4 VA Boston Healthcare System, US Department of Veteran Affairs, Boston, Massachusetts; 5 VA Bedford Healthcare System, US Department of Veteran Affairs, Bedford, Massachusetts; 6 Framingham Heart Study, Boston University Chobanian and Avedisian School of Medicine, Boston, Massachusetts; 7 Biostatistics and Epidemiology Data Analytics Center, Boston University School of Public Health, Boston, Massachusetts; 8 Concussion Legacy Foundation, Boston, Massachusetts; 9 Department of Pathology and Laboratory Medicine, Boston University Chobanian and Avedisian School of Medicine, Boston, Massachusetts; 10 Department of Neurology, Boston University Chobanian and Avedisian School of Medicine, Boston, Massachusetts; 11 Department of Rehabilitation and Human Performance, Brain Injury Research Center, Icahn School of Medicine at Mount Sinai, New York, New York; 12 Department of Neurology, Icahn School of Medicine at Mount Sinai, New York, New York; 13 Department of Pathology, Molecular and Cell-Based Medicine, Icahn School of Medicine at Mount Sinai, New York, New York; 14 Braintree Rehabilitation Hospital, Braintree, Massachusetts; 15 Department of Rehabilitation Medicine, Harvard Medical School, Boston, Massachusetts; 16 Department of Biomedical Engineering, Boston University College of Engineering, Boston, Massachusetts; 17 Department of Psychiatry, Boston University Chobanian and Avedisian School of Medicine, Boston, Massachusetts; 18 Department of Radiology, Boston University Chobanian and Avedisian School of Medicine, Boston, Massachusetts; 19 Department of Anatomy and Neurobiology, Boston University Chobanian and Avedisian School of Medicine, Boston, Massachusetts; 20 Department of Epidemiology, Boston University School of Public Health, Boston, Massachusetts; 21 Department of Neurosurgery, Emerson Hospital, Concord, Massachusetts; 22 Department of Neurosurgery, Boston University Chobanian and Avedisian School of Medicine, Boston, Massachusetts

## Abstract

**Importance:**

Chronic traumatic encephalopathy (CTE) is a neurodegenerative tauopathy associated with repetitive head impacts (RHIs). Prior research suggests a dose-response association between American football play duration and CTE risk and severity, but this association has not been studied for ice hockey.

**Objective:**

To investigate associations of duration of ice hockey play with CTE diagnosis and severity, functional status, and dementia.

**Design, Setting, and Participants:**

This cross-sectional study was conducted among male brain donors in the Understanding Neurological Injury and Traumatic Encephalopathy and Framingham Heart Study Brain Banks whose primary RHI exposure was from ice hockey. Donors died, brains were donated, and data were collected between July 1997 and January 2023. Data analysis was conducted from January 2023 to May 2024.

**Exposures:**

Ice hockey years played as an RHI proxy.

**Main Outcomes and Measures:**

CTE neuropathological diagnosis, cumulative phosphorylated tau (ptau) burden across 11 brain regions commonly affected in CTE, informant-reported Functional Activities Questionnaire (FAQ) score at death, and consensus dementia diagnosis were assessed.

**Results:**

Among 77 male donors (median [IQR] age, 51 [33-73] years), 42 individuals (54.5%) had CTE, including 27 of 28 professional players (96.4%). CTE was found in 5 of 26 donors (19.2%) who played fewer than 13 years, 14 of 27 donors (51.9%) who played 13 to 23 years, and 23 of 24 donors (95.8%) who played more than 23 years of hockey. Increased years played was associated with increased odds for CTE (odds ratio [OR] per 1-year increase, 1.34; 95% CI, 1.15-1.55; *P* < .001) and with increased ptau burden (SD increase per 1-year increase = 0.037; 95% CI, 0.017-0.057; *P* < .001) after adjusting for age at death, other contact sports played, age of first hockey exposure, concussion count, and hockey position. Simulation demonstrated that years played remained associated with CTE when years played and CTE were both associated with brain bank selection across widely ranging scenarios (median [full range] OR across all simulations, 1.34 [1.29-1.40]). Increased ptau burden was associated with FAQ score (β_standardized_ = 0.045; 95% CI, 0.021-0.070; *P* < .001) and dementia (OR per SD increase, 1.12; 95% CI, 1.01-1.26; *P* = .04) after adjusting for age at death, other contact sports played, hockey years played, enforcer status, age of first hockey exposure, concussion count, and hockey position.

**Conclusions and Relevance:**

In this study of male former ice hockey players, a dose-response association was observed between hockey years played and risk and severity of CTE. Simulation suggested that brain bank selection may not bias the magnitude of outcomes in the association.

## Key Points

**Question:**

Is duration of ice hockey play associated with the presence and severity of chronic traumatic encephalopathy (CTE) pathology?

**Findings:**

In this cross-sectional study of 77 male brain donors who were amateur and professional ice hockey players, 27 of 28 professional players (96.4%) had CTE pathology and there was a dose-response association between years of ice hockey played and the presence and severity of CTE pathology, with the odds of having CTE and cumulative phosphorylated tau burden increasing per each additional year played.

**Meaning:**

This study found that risk for CTE may cumulatively increase with each additional year of ice hockey played, as with American football.

## Teaser

This cross-sectional study investigates the association of duration of ice hockey play with chronic traumatic encephalopathy presence and severity among male brain donors.

## Introduction

Chronic traumatic encephalopathy (CTE) is a neurodegenerative tauopathy associated with exposure to repetitive head impacts (RHIs).^1,2,3,4,5,6,7,8,9^ CTE has been most commonly diagnosed in individuals exposed to RHIs from contact sports.^1,5,8,10,11^ CTE can be definitively diagnosed only via postmortem neuropathological examination. Criteria for CTE diagnosis were defined and refined by a consensus panel of neuropathologists organized by the National Institute of Neurological Disorders and Stroke and the National Institute of Biomedical Imaging and Bioengineering (NINDS-NIBIB).^12,13^ This panel concluded that the pattern of neuronal, perivascular phosphorylated tau (ptau) pathology in CTE is distinct from other tauopathies.^13^

A growing literature supports a dose-response association between the amount of contact sport play and CTE pathology occurrence and severity. Most neuropathologically diagnosed CTE cases have been in former contact sport athletes, predominantly elite American football players.^1,2,3^ Among 266 former American football players whose highest level of play spanned from youth to professional levels, odds for developing CTE were found to increase by 30% with each additional year played, suggesting a dose-response association.^3^ Among football players with CTE, odds for developing severe CTE were found to increase by 14% with each additional year played. Among 31 rugby union players who came to autopsy, 21 individuals had CTE and those with CTE played significantly longer (mean, 21.5 vs 12.1 years).^14^

Fewer CTE cases have been described in former ice hockey players, and the nature of the association between RHIs from ice hockey play and CTE risk is not well understood. Nonetheless, ice hockey players have substantial RHI exposure from checking and fighting. Among youth hockey players, there is a 3-fold increased risk for head injuries in leagues permitting body checking compared with leagues not permitting body checking.^15^ Furthermore, enforcers, also known as goons or fighters, have an unofficial role at elite levels to react aggressively to perceived violent or dirty play, often initiating physical fights against offenders. National Hockey League (NHL) enforcers die a mean of 10 years earlier than their nonenforcer counterparts.^16^ A 2021 study^17^ of 11 elite ice hockey players who came to autopsy found that 6 individuals had CTE. In an analysis combining these ice hockey players with 24 Canadian Football League players, the authors did not find an association between position played or career duration and CTE; however, due to the small sample size, it was not possible to analyze each sport individually. Consequently, associations of lifelong duration of ice hockey play and enforcer status with CTE remain largely unexamined. Here, we tested the hypothesis that increasing duration of ice hockey play and enforcer status would be associated with a corresponding increase in CTE risk and severity. Additionally, we used inverse probability weighting (IPW) and simulation to adjust for potential selection bias in reported estimated associations.^18^ To verify that CTE neuropathological changes were clinically meaningful, we tested the association of CTE severity with dementia diagnosis and an informant-reported scale of daily function that has been previously associated with CTE pathology.^19^

## Methods

The institutional review board at Boston University’s Medical Campus and the VA Bedford Healthcare System approved all relevant research activities for this cross-sectional study. Next of kin provided informed consent for brain donation. This report followed the Strengthening the Reporting of Observational Studies in Epidemiology (STROBE) reporting guideline for cross-sectional studies. See the eMethods in Supplement 1 for complete methods.

### Donor Recruitment

All study brain donors were part of the Understanding Neurological Injury and Traumatic Encephalopathy (UNITE) or Framingham Heart Study (FHS) brain banks. Criteria for inclusion in both studies have been previously described.^2,3,20,21^ In this study, our target population was male athletes whose primary sport was ice hockey, with the highest level of play spanning youth to professional play. We included donors from both brain banks whose primary contact sport exposure (defined subsequently) came from organized ice hockey play. We included donors from both studies to increase the sample size and improve representation across the spectrum of duration of ice hockey play. See eFigure 1 in Supplement 1 for the inclusion and exclusion flowchart. Race and ethnicity were informant reported. Race and ethnicity were assessed because they are factors commonly associated with brain donation. Race options in the surveys were American Indian or Alaska Native, Asian, Black or African American, Native Hawaiian or Pacific Islander, White, unknown, and other (specify in text box). Ethnicity options in the surveys were Hispanic and not Hispanic.

### Contact Sport and Traumatic Brain Injury History

Retrospective data collection from informants was similar for both brain banks. For each contact sport exposure (hockey or otherwise), informants provided ages the donor began and stopped playing, levels the donor played (youth, high school, college, juniors or semiprofessional, and professional), and positions and total years the donor played at each level. For donors who played multiple sports, we used the highest level played to define primary sport exposure. Although only donors whose primary sport was hockey were ultimately included, playing additional contact sports beyond hockey was incorporated into statistical analyses. We defined ice hockey play duration as the total years of organized ice hockey play.^22,23,24,25^ We excluded years played in informal men’s leagues, which usually do not allow checking. However, we included youth play given that checking may occur (albeit not in all leagues and we did not confirm donor-specific checking during youth play). For donors who played hockey at the professional level, position and years played were verified using a public database.^26^ Informants were asked whether during their playing career, donors were considered enforcers, an unofficial role whereby players are expected to engage in fights and respond aggressively to physical play by the opposition.

### Clinical Evaluation

For the UNITE brain bank, previously detailed methods for retrospective clinical data collection and comprehensive medical record review were followed for all brain donors.^1,20^ Instrumental activities of daily living were assessed using the Functional Activities Questionnaire (FAQ),^27^ completed by informants. Clinicians with expertise in neurodegenerative disease (K.D.O.C., B.D., D.H.D., L.E.G., D.I.K., N.W.K., R.C.C., R.A.S., M.L.A., and J.M.) reviewed all cases to reach consensus on a dementia diagnosis based on modified *Diagnostic and Statistical Manual of Mental Disorders* (Fourth Edition, Text Revision) criteria. Clinicians and clinical research assistants were blinded to the neuropathological examination and findings. For the FHS brain bank, participants suspected of having cognitive impairment were prospectively brought to a consensus meeting in life, during which it was determined whether the participant met criteria for dementia.^28^

### Pathological Evaluation

Neuropathological evaluation for both brain banks followed similar, previously established protocols and was carried out by the same neuropathologists (V.E.A., B.R.H., T.D.S., and A.C.M.). CTE neuropathological diagnosis was based on NINDS-NIBIB criteria.^13^ Additionally, neuropathologists assigned semiquantitative ptau burden measures across 11 regions implicated in CTE (dorsolateral frontal cortex, inferior orbital frontal cortex, superior temporal cortex, inferior parietal cortex, hippocampus CA1, hippocampus CA2, hippocampus CA4, entorhinal cortex, amygdala, substantia nigra, and locus coeruleus) on a 0 to 3 severity scale.

### Imputation of Missing Semiquantitative Ptau Burden

Missing semiquantitative ptau values across the 11 brain regions were imputed using multiple imputation by chained equations. Cumulative ptau burden was calculated by summing semiquantitative ptau measures (including imputed and directly measured values) across the 11 brain regions. See eTable 1 in Supplement 1 for semiquantitative ptau burden missingness across the 11 regions.

### Statistical Analysis

We tested the association of duration of ice hockey play with 2 main neuropathological outcomes pertaining to CTE: CTE diagnosis and cumulative ptau burden. We used binary logistic regression to estimate the association of duration played with CTE diagnosis and linear regression to estimate the association of duration played with cumulative ptau burden. All primary models testing associations between CTE diagnosis and cumulative ptau burden included as covariates age at death, dichotomized participation in other contact sport play, position of hockey play (offense vs other), age of first exposure to hockey play, and concussion count given that these factors have been hypothesized to be associated with CTE.^1,2,3,25^ See the eMethods in Supplement 1 for the description of 5 additional sensitivity analyses.

We plotted a receiver operating characteristic curve to observe how well our model classified CTE diagnosis. We identified thresholds of duration of hockey played that corresponded to negative and positive likelihood ratios (LRs) closest to 0.1 and 10, respectively, values that may produce sizable and often conclusive shifts from pretest to posttest probability. We also identified a threshold that maximizes the mean of sensitivity and specificity of duration of hockey played together, based on the Youden index, concordance probability, and point closest to corner.^29^

To quantify conditions under which selection bias could invalidate our findings, we conducted simulation analyses using methods previously described.^3^ We focused on associations of duration of hockey played and CTE with selection. We assumed the probability of selection, P_i_(S), is a function of duration played (D), CTE diagnosis (C), and their cross product (DC) in a logistic regression model and that *K* is the log odds of selection when D, C, and DC are not associated with selection:

log P_i_(S)/1 − P_i_(S) = β_D_D + β_C_C + β_DC_DC + *K*.

We set *K* = −1.17, the log odds of selection into a brain bank from a community-based study.^30^ For each individual, *i*, we calculated P_i_(S) for a range of values of β_D_, β_C_, and β_DC_ (0-3 in intervals of 0.1) and used 1/P_i_(S) as a weight in IPW analyses evaluating the association of duration played with CTE diagnosis. We limited β_D_, β_C_, and β_DC_ to nonnegative values based on the assumption that greater duration played and the presence of CTE pathology may increase but would not decrease brain donation probability. For each set of values of β_D_, β_C_, and β_DC_, we estimated the association of duration played with CTE diagnosis. We repeated this approach for cumulative ptau burden.

Lastly, we tested the association of cumulative ptau burden with dementia diagnosis and FAQ scores to verify whether CTE neuropathological changes were clinically meaningful in ice hockey players. We used binary logistic and linear regression for models with dementia and FAQ scores, respectively, as outcomes. Models were adjusted for age at death, concussion count, dichotomized participation in other contact sport play, position of hockey play, age of first exposure to hockey play, other contact sports played, duration of ice hockey play, and enforcer status. Data were analyzed from January 2023 to May 2024. Statistical significance was set at *P* <.05, with all statistical tests 2-sided. All analyses were performed using SPSS statistical software version 28 (IBM) and RStudio statistical software version 2023.06 .0+421 (RStudio). All data were collected and stored using REDCap version 13.8.1.

## Results

**Figure 1.**
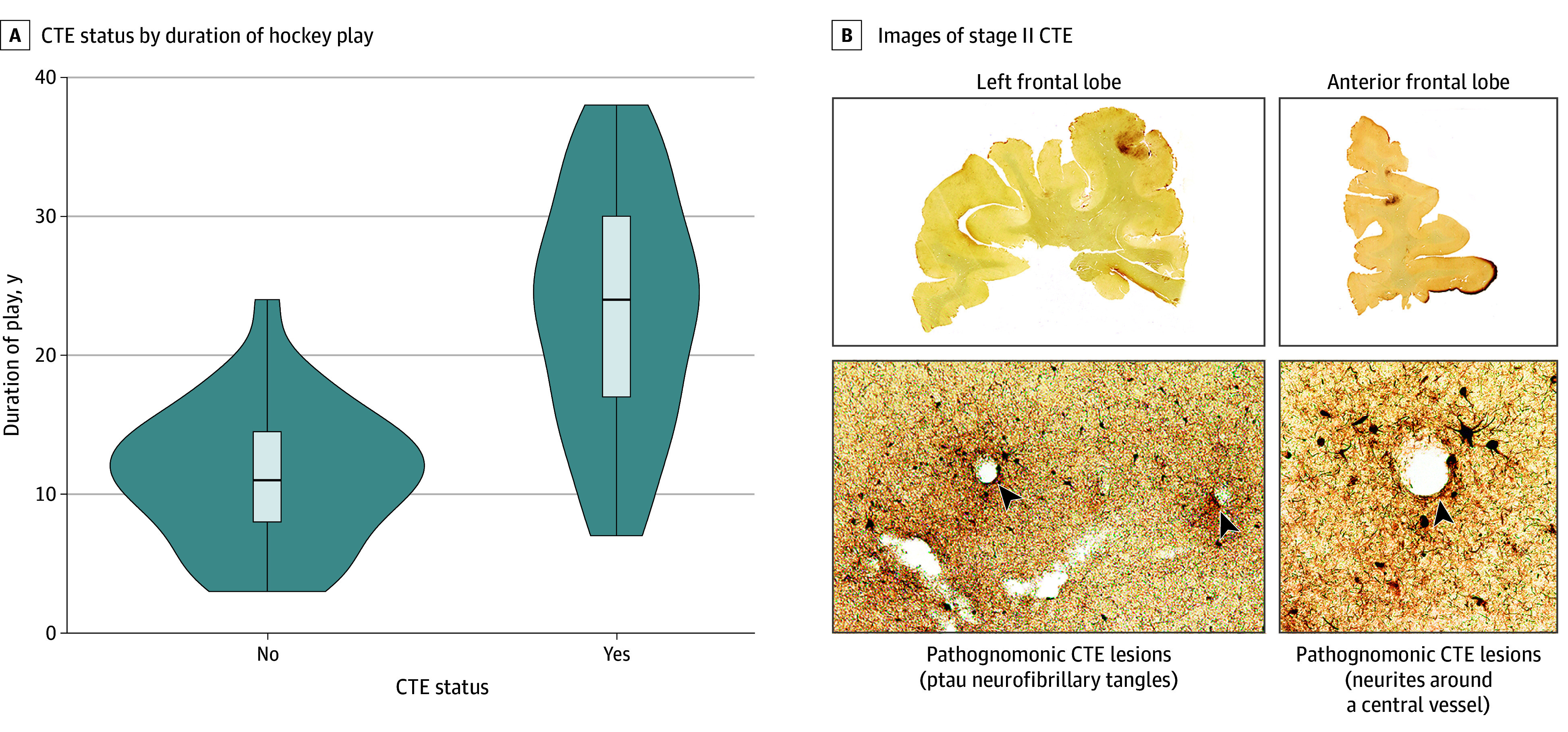
Distribution of Duration of Ice Hockey Play and Chronic Traumatic Encephalopathy (CTE) Photomontage A, Violin plots display the distribution of duration of ice hockey play stratified by CTE diagnosis. B, The photomontage of stage II CTE shows hemispheric 50-μm tissue sections immunostained with AT8. Positive phosphorylated tau (ptau) immunostaining appears dark brown, showing a large CTE lesion in the left frontal lobe and right anterior frontal lobe. Pathognomonic CTE lesions (arrows) consisting of ptau neurofibrillary tangles and neurites around a central vessel are shown. Magnification is ×200.

Among 77 donors (median [IQR; SD] age, 51 [33-73; 22.8] years; 77 White [100%]), 42 donors (54.5%) were diagnosed with CTE and 35 donors (45.4%) were not diagnosed with CTE. The most common causes of death were suicide (CTE: 12 donors [28.6%]; no CTE: 10 donors [28.6%]) and neurodegenerative disease (CTE: 11 donors [26.2%]; no CTE: 8 donors [22.9%]) (Table 1; eTable 2 in Supplement 1). There were a mean (SD) of 2 (1.4) informants per donor, and informants knew the donors for a mean (SD) of 38.2 (17.1) years. Informants included 27 spouses (35.1%), 27 parents (35.1%), 12 children (15.6%), and 11 siblings (14.3%). Among 28 former professional ice hockey players, 27 players (96.4%) were diagnosed with CTE, including 18 of 19 former NHL players (94.7%), 13 of 28 college, juniors, and semiprofessional players (46.4%), and 2 of 21 youth and high school players (9.5%). In total, 18 of 22 enforcers (81.8%) were diagnosed with CTE. The 22 enforcers in the study had a mean (SD) 22.9 (8.5) years of play. NHL enforcers had a significantly more mean (SD) penalty minutes (2.15 [1.15] vs 0.72 [0.29] minutes; *P* = .002) and fights (0.20 [0.19] vs 0.02 [0.01] fights; *P* = .01) per game than NHL nonenforcers. CTE was diagnosed in 5 of 26 donors (19.2%) who played fewer than 13 years, 14 of 27 donors (51.9%) who played between 13 and 23 years, and 23 of 24 donors (95.8%) who played more than 23 years. Of donors diagnosed with CTE, 11 donors (26.2%) had stage I CTE, 11 donors (26.2%) had stage II CTE, 13 donors (31.0%) had stage III CTE, and 7 donors (16.7%) had stage IV CTE. Among all 77 donors, 28 individuals (36.4%) played other contact sports, including 22 individuals (28.6%) who played American football (Table 1). See eTable 2 in Supplement 1 for demographic and exposure variables stratified by CTE diagnosis. Figure 1A displays the distribution of duration played in years stratified by CTE diagnosis.

**Table 1.** Sample Characteristics

Characteristic	Donors (%)			
	<13 y of Play (n = 26)	13-23 y of Play (n = 27)	>23 y of Play (n = 24)	Total (N = 77)
Demographics				
Male	26 (100)	27 (100)	24 (100)	77 (100)
White	26 (100)	27 (100)	24 (100)	77 (100)
Age at death, median (IQR) [range], y	47 (30-68) [13-91]	52 (27-64) [19-88]	57 (41-80) [28-89]	51 (33-73) [13-91]
Cause of death				
Neurodegenerative disease	7 (26.9)	4 (14.8)	8 (33.3)	19 (24.7)
Cardiovascular disease	2 (7.7)	5 (18.5)	1 (4.2)	8 (10.4)
Suicide	10 (38.5)	7 (25.9)	5 (20.8)	22 (28.6)
Cancer	2 (7.7)	1 (3.7)	3 (12.5)	6 (7.8)
Motor neuron disease	0	0	2 (8.3)	2 (2.6)
Accidental overdose	2 (7.7)	3 (11.1)	2 (8.3)	7 (9.1)
Other^a^	3 (11.5)	4 (14.8)	3 (13.0)	10 (13.0)
Unknown	0	3 (11.1)	0	3 (3.9)
Clinical outcomes				
Dementia	7 (26.9)	8 (29.6)	13 (54.2)	28 (36.4)
FAQ score, mean (SD)	5.33 (9.28)	8.63 (11.31)	13.22 (12.31)	9.34 (11.45)
Repetitive head impact exposure				
Concussion count				
No. with data	25	22	22	69
Median (IQR)	10 (0-28)	22 (11-60)	30 (15-100)	20 (10-50)
AFE to ice hockey, median (IQR), y	8 (6-14)	7 (5-8)	5 (4-10)	7 (5-10)
Duration of play, mean (SD), y	8.4 (2.9)	15 (2.9)	28.5 (4.6)	17.7 (9.2)
Highest level of play				
Youth	3 (11.5)	0	0	3 (3.8)
High school	15 (57.7)	3 (11.1)	0	18 (23.4)
College	5 (19.2)	9 (33.3)	1 (4.2)	15 (19.5)
Juniors or semiprofessional	3 (11.5)	8 (29.6)	2 (8.3)	13 (16.9)
Professional	0	7 (25.9)	21 (87.5)	28 (36.4)
Primary position at highest level of play				
Forward	13 (50.0)	15 (55.6)	17 (70.8)	45 (58.4)
Defenseman	8 (30.8)	9 (33.3)	6 (25.0)	23 (29.9)
Goaltender	0	1 (3.7)	0	1 (1.3)
Unknown or undisclosed	5 (19.2)	2 (7.4)	1 (4.2)	8 (10.4)
Enforcer	2 (7.7)	9 (33.3)	11 (45.8)	22 (28.6)
Military veteran	12 (46.2)	4 (14.8)	1 (4.2)	17 (22.1)
Other contact sport play	14 (53.8)	7 (25.9)	7 (29.2)	28 (36.4)
Neuropathology^b^				
Brain weight, mean (SD), g	1397.3 (149.1)	1405.5 (173.7)	1366.6 (180.1)	1391.3 (167.1)
CTE stage				
I	3 (11.5)	4 (14.8)	4 (16.7)	11 (14.3)
II	1 (3.8)	3 (11.1)	7 (29.2)	11 (14.3)
III	0	4 (14.8)	9 (37.5)	13 (16.9)
IV	1 (3.8)	3 (11.1)	3 (12.5)	7 (9.1)
Neurodegenerative diagnosis				
Alzheimer disease^c^	3 (11.5)	3 (11.1)	6 (25.0)	12 (15.6)
Lewy body disease	2 (7.7)	5 (18.5)	7 (29.2)	14 (18.2)
Motor neuron disease	0	0	2 (8.3)	2 (2.6)
FTLD-tau	0	1 (3.7)	1 (4.2)	2 (2.6)
FTLD-TDP-43	0	0	1 (4.2)	1 (1.3)
Neuritic plaques, CERAD score				
No. with data	26	27	22	75
None (C0)	20 (76.9)	20 (74.1)	14 (63.6)	54 (72.0)
Sparse (C1)	5 (19.2)	3 (11.1)	5 (22.7)	13 (17.3)
Moderate (C2)	1 (3.8)	3 (11.1)	3 (13.6)	7 (9.3)
Frequent (C3)	0	1 (3.7)	0	1 (1.3)
Diffuse plaques, CERAD score				
No. with data	26	27	22	75
None	18 (69.2)	18 (66.7)	12 (54.5)	48 (64.0)
Sparse	2 (7.7)	2 (7.4)	4 (18.2)	8 (10.7)
Moderate	2 (7.7)	2 (7.4)	4 (18.2)	8 (10.7)
Frequent	4 (15.4)	5 (18.5)	2 (9.1)	11 (14.7)
Thal phase				
No. with data	19	25	22	66
0 (A0)	15 (78.9)	18 (72.0)	12 (54.5)	45 (68.2)
1-2 (A1)	2 (10.5)	1 (4.0)	2 (9.1)	5 (7.6)
3 (A2)	0	2 (8.0)	4 (18.2)	6 (9.1)
4-5 (A3)	2 (10.5)	4 (16.0)	4 (18.2)	10 (15.2)
Braak stage				
No. with data	26	27	22	75
0 (B0)	18 (69.2)	13 (48.1)	5 (22.7)	36 (48.0)
1-2 (B1)	3 (11.5)	5 (18.5)	4 (18.2)	12 (16.0)
3-4 (B2)	2 (7.7)	4 (14.8)	7 (31.8)	13 (17.3)
5-6 (B3)	3 (11.5)	4 (14.8)	6 (27.3)	13 (17.3)
White matter rarefaction, moderate to severe, No./total No. (%)	7/25 (28)	7/27 (25.9)	8/22 (36.4)	22/74 (29.7)
Atherosclerosis, moderate to severe, No./total No. (%)	5/26 (19.2)	4/27 (14.8)	4/22 (18.2)	13/75 (17.3)
Arteriolosclerosis, moderate to severe, No./total No. (%)	7/26 (26.9)	6/27 (22.2)	7/22 (31.8)	20/75 (26.7)
TDP-43 inclusions, No./total No. (%)	1/19 (5.2)	4/25 (16.0)	7/23 (30.4)	12/67 (15.6)
Hippocampal sclerosis, No./total No. (%)	0/19	1/25 (4.0)	3/23 (13.0)	4/67 (5.2)
Semiquantitative ratings of ptau severity, 0-3 scale, mean (SD)				
Dorsolateral frontal cortex	0.52 (1.01)	1.07 (1.07)	2.19 (0.81)	1.19 (1.18)
Inferior orbital frontal cortex	0.33 (0.97)	1.00 (1.08)	1.64 (0.90)	1.03 (1.11)
Superior temporal cortex	0.52 (1.12)	0.89 (1.12)	1.78 (0.95)	1.04 (1.19)
Inferior parietal cortex	0.36 (0.91)	0.85 (1.13)	1.39 (1.23)	0.86 (1.16)
CA1	0.58 (1.14)	1.15 (1.20)	1.65 (1.27)	1.1 (1.25)
CA2	0.45 (0.96)	0.72 (1.06)	1.23 (1.02)	0.78 (1.05)
CA4	0.29 (0.85)	0.72 (1.10)	1.26 (1.01)	0.8 (1.07)
Entorhinal	0.76 (1.09)	1.11 (1.16)	2.00 (1.04)	1.24 (1.19)
Amygdala	0.76 (1.20)	1.00 (1.17)	1.82 (1.01)	1.15 (1.21)
Substantia nigra	0.32 (0.75)	0.81 (1.00)	1.00 (1.04)	0.79 (1.28)
Locus coeruleus	0.61 (0.61)	1.54 (1.22)	1.95 (0.89)	1.39 (1.1)

Abbreviations: AFE, age of first exposure; CERAD, Consortium to Establish a Registry for Alzheimer Disease; CTE, chronic traumatic encephalopathy; FAQ, Functional Activities Questionnaire; FTLD, frontotemporal lobar degeneration; ptau, phosphorylated tau; TDP-43, TAR DNA-binding protein 43.

^a^ Other causes of death included infection (2 donors), motor vehicle accidents (2 donors), alcoholic cirrhosis, COVID-19, stroke, acute necrotizing pancreatitis, and other unspecified causes (2 donors).

^b^ Sample sizes for neuropathological outcomes ranged from 66 to 77 donors because certain measures were not assessed for all donors.

^c^ National Institute on Aging (NIA)-Reagan level intermediate or high.

Figure 1B shows representative images in a donor with stage II CTE neuropathology. Table 1 shows frequencies of comorbid pathologies and measures of semiquantitative ptau burden across regions in donors stratified by duration of play tertiles (<13 years, 13-23 years, and >23 years). Semiquantitative ptau burden scores across all 11 regions increased with each increasing tertile played. Frequencies of comorbid pathologies and measures of semiquantitative ptau burden across regions stratified by CTE diagnosis are shown in eTable 2 in Supplement 1.

**Figure 2.**
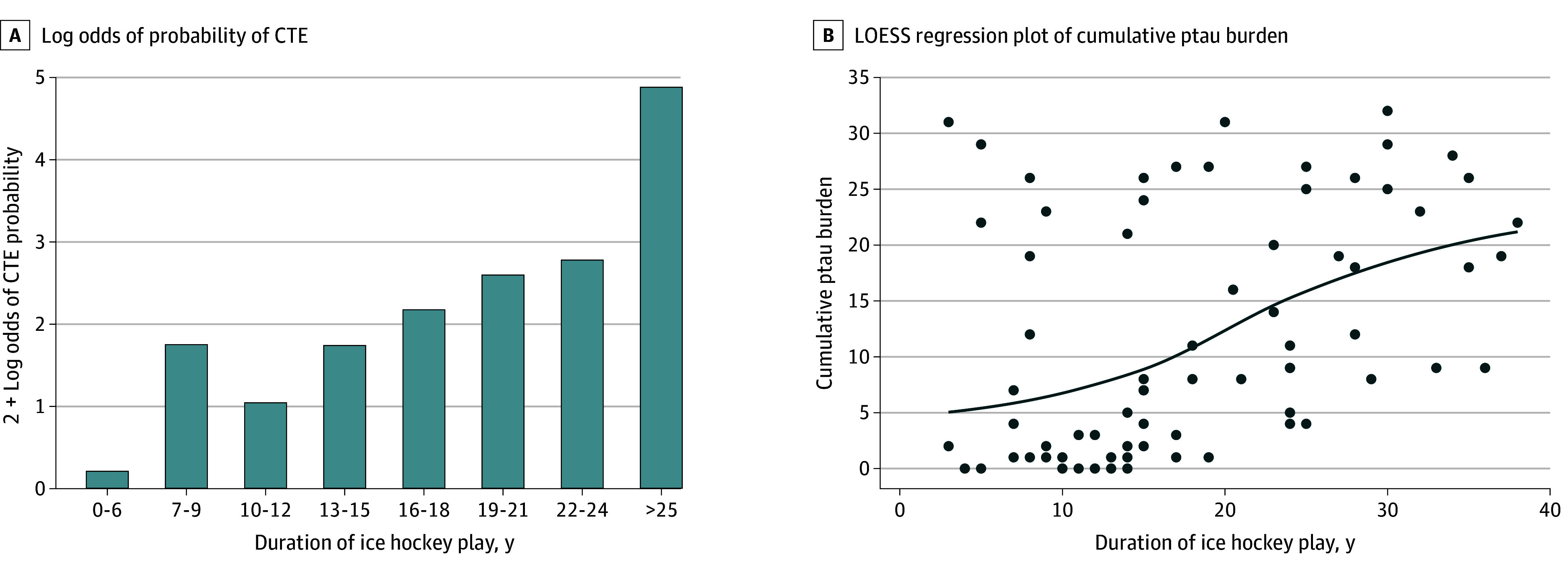
Evaluating Linearity Assumption A, The bar graph shows 2 + the log odds of the probability of chronic traumatic encephalopathy (CTE) by duration of hockey played in 3-year intervals. For durations of 0 to 6 years and 25 or more years, the log odds of CTE probability could not be calculated because no donors had CTE (for 0-6 years) or all donors had CTE (≥25 years). For the figure, log odds were calculated with probabilities of 1 of 7 CTE cases for durations of 0 to 6 years and 18 of 19 CTE cases for durations of 25 or more years. There were 0 of 6 donors, 4 of 11 donors (36.4%), 1 of 10 donors (10.0%), 5 of 14 donors (35.7%), 3 of 5 donors (60.0%), 4 of 5 donors (80.0%), 6 of 7 donors (85.7%), and 19 of 19 donors (100%) with CTE at 0 to 6, 7 to 9, 10 to 12, 13 to 15, 16 to 18, 19 to 21, 22 to 24, and 25 or more years of duration, respectively. B, The locally estimated scatterplot smoothing (LOESS) regression plot shows cumulative phosphorylated tau (ptau) burden by duration played.

In models adjusted for age at death, concussion count, participation in other contact sport play, position of hockey play, and age of first exposure to hockey play, a dose-response association was observed between increasing duration of ice hockey play and CTE diagnosis (odds ratio [OR] per 1-year increase in hockey play, 1.34; 95% CI, 1.15-1.55; *P* < .001) Table 2). There was a dose-response association between increasing duration of ice hockey play and greater cumulative semiquantitative ptau burden, with each additional year of ice hockey play corresponding to a 0.037 SD increase in cumulative ptau burden (95% CI, 0.017-0.057; *P* < .001) (Table 2). Primary models satisfied the linearity assumption. Specifically, for CTE diagnosis, the Hosmer-Lemeshow χ^2^ test was not significant, suggesting that models fit the observed data well, and the bar graph of log odds of the probability of CTE by duration played in 3-year intervals demonstrated a linear pattern (Figure 2A). For ptau burden, a locally estimated scatterplot smoothing regression plot depicting the cumulative ptau burden by duration played demonstrated a linear pattern (Figure 2B).

**Table 2.** Association Between Duration Played and CTE Neuropathology

Factor	CTE neuropathology^a^	*P* value
**Duration played** ^b^		
CTE diagnosis, OR per 1-y duration (95% CI)	1.34 (1.15 to 1.55)	<.001
Cumulative ptau burden, β, SD per 1-y duration (95% CI)	0.037 (0.017 to 0.057)	<.001
**Duration played and enforcer status**		
CTE diagnosis		
OR per 1-y duration (95% CI)	1.30 (1.12 to 1.52)	<.001
Enforcer status, OR (95% CI)	2.03 (0.39 to 10.62)	.40
Cumulative ptau burden		
β, SD per 1-y duration (95% CI)	0.029 (0.01 to 0.05)	.01
Enforcer status, β (95% CI), SD	0.35 (−0.05 to 0.74)	.08
**Simulation assessing association between duration played and CTE neuropathology** ^c^		
CTE diagnosis, median OR per 1-y duration (full range)	1.34 (1.29 to 1.40)	NA
Cumulative ptau burden, β, median SD per 1-y duration (full range)	0.039 (0.026 to 0.046)	NA

Abbreviations: CTE, chronic traumatic encephalopathy; NA, not applicable; OR, odds ratio; ptau, phosphorylated tau.

^a^ All models were adjusted for age at death, non–ice hockey contact sport play, age of first exposure to ice hockey play, position of play, coded vs not coded as forward (wingers and centers), and total concussions.

^b^ Separate models were run for each outcome. The sample size for all analyses is 77 donors (42 donors with and 35 donors without CTE).

^c^ For both simulations, median OR magnitudes were similar to those for the actual brain bank sample and the full range of association magnitudes were never null (ie, OR = 1 or SD increase = 0). Simulation assesses ORs with a range of nonnegative values for selection parameters for duration played (β_D_; ie, log odds of brain donation for each additional year played when CTE pathology is absent), CTE diagnosis (β_C_; ie, log odds of brain donation when CTE pathology is present compared with absent when duration played is approaching zero), and duration played × CTE diagnosis cross product (β_DC_; ie, additional log odds of brain donation for each additional year played beyond β_D_, when CTE pathology is present).

Table 2 shows models that included duration played and enforcer status. There was no association between being an enforcer and having CTE (OR, 2.03; 95% CI, 0.39 to 10.62; *P* = .40) or SD increase in cumulative ptau burden (0.35; 95%CI, −0.05 to 0.74; *P* = .08). For models including enforcer status, outcome magnitude in the association of duration played with CTE diagnosis and cumulative ptau burden were reduced but were still significant (Table 2). Similar outcome magnitudes to the primary analyses were observed when only donors in the UNITE Brain Bank were included, when donors who played 5 or more years of American football were removed, and when models were also adjusted for non-CTE tauopathy diagnosis (eTable 3 in Supplement 1). When excluding age at death from models (eTable 3 in Supplement 1), outcome magnitude was similar for CTE diagnosis and was larger for cumulative ptau burden, suggesting that age at death may be a mediator.

The area under the receiver operating characteristic curve depicting estimated probabilities for duration of ice hockey play and CTE diagnosis was 0.89 (95% CI, 0.81-0.96), suggesting that duration of ice hockey play may be a good classifier of CTE diagnosis (eFigure 2 in Supplement 1). Donors with CTE were roughly 10 times as likely to have played at least 18 years of hockey (positive likelihood ratio = 10.2; sensitivity = 0.70; specificity = 0.93), and were roughly one-tenth as likely to have played fewer than 7.5 years of hockey (negative likelihood ratio = 0.1; sensitivity = 0.98; specificity = 0.23) compared with donors who did not have CTE. There was 1 donor with CTE who played fewer than 7.5 years, while 2 donors without CTE played more than 18 years. We found the optimal cut point to maximize sensitivity and specificity to be 16 years of ice hockey play (sensitivity = 0.76; specificity = 0.89).

We conducted simulation analyses assessing how brain bank selection based on duration of hockey played, CTE diagnosis, and their cross product may have biased the duration of hockey played and CTE pathology association. Across all nonnegative selection regression coefficients β_D_, β_C_, and β_DC_, the median (range) selection-adjusted OR of having CTE for each additional year played was 1.34 (1.29-1.40) (Table 2). Across all nonnegative selection regression coefficients β_D_, β_C_, and β_DC_ the median (range) selection-adjusted SD increase in cumulative ptau burden for each additional year played was 0.039 (0.026-0.046) (Table 2). In each simulation, IPWs ranged from 1 to 4.23, demonstrating that there were no extreme weights.

Additional models tested the association of cumulative ptau burden with dementia diagnosis and FAQ scores, adjusting for age at death, concussion count, dichotomized participation in other contact sport play, position of hockey play (offense vs other), age of first exposure to hockey play, other contact sports played, duration of ice hockey play, and enforcer status. Each unit increase in cumulative ptau burden corresponded to an increase in dementia odds (OR, 1.12; 95% CI, 1.01-1.26; *P* = .04), Table 3). Each unit increase in cumulative ptau burden corresponded to a 0.045 SD increase in FAQ score (95%CI, 0.021-0.070; *P* < .001), Table 3).

**Table 3.** Association of Cumulative Ptau Burden With Dementia Diagnosis and FAQ Scores

Outcome	Outcome per 1-SD ptau burden increase (95% CI)^a^	*P* value
Dementia diagnosis, OR	1.12 (1.01-1.26)	.04
FAQ score, SD increase	0.045 (0.021-0.07)	<.001

Abbreviations: FAQ, Functional Activities Questionnaire; OR, odds ratio; ptau, phosphorylated tau.

^a^ Separate models were run for each outcome. Models were adjusted for age at death, duration of ice hockey play, enforcer status, non–ice hockey contact sport play, age of first exposure to ice hockey play, position of play, coded vs not coded as forward (wingers and centers), and total concussions.

## Discussion

In this cross-sectional study of brain donors whose primary RHI exposure came from ice hockey play, we investigated the association of duration of ice hockey play in years with CTE risk and cumulative ptau burden. We found that duration played was associated with CTE risk, with odds of having CTE increasing by 34% per year played. Duration played was likewise associated with increased cumulative ptau burden. Simulation demonstrated that years played remained associated with CTE presence and severity when years played and CTE were both associated with brain bank selection across widely ranging scenarios.

To our knowledge, this is the first study to show a dose-response association between ice hockey play and CTE diagnosis, as well as cumulative ptau burden. A similar association has been noted among former American football players and rugby union players.^3,14^ Career length has also been associated with all-cause neurodegenerative disease diagnosed in life in association football players.^3,31^ These findings provide added evidence that increasing RHI exposure from contact sport play, regardless of whether symptoms are present at the time of impact, is a risk factor associated with CTE.

Analyses were limited to individuals whose primary RHI exposure source was ice hockey play, although we still allowed for other contact sport play secondarily. We included other contact sport play as a covariate given that American football and other non–ice hockey sport play may confound the association between ice hockey play and CTE. Concurrent nonhockey contact sport play could be associated with less hockey play and with increased risk for CTE.

We found that 18 of 22 enforcers had CTE. Notably, most enforcers played at high levels, playing a mean (SD) of 22.9 (8.5) years. In models that included enforcer status and duration played, only duration played remained associated with CTE neuropathology, although enforcers were still more likely to have CTE. Including enforcer status in the model reduced the outcome magnitude in the association of duration played, suggesting that enforcer status may marginally mediate the association between duration played and CTE pathology, although most of the association appears to be independent of enforcer status. Predictably, NHL enforcers in our study had a higher mean number of penalty minutes and fights per game compared with nonenforcer NHL players. These findings give some credence to fighting portending additional risk for CTE, although a larger study with additional power is needed, and our findings lend support to recent efforts to reduce fighting across all hockey levels to protect players.^32^

Brain donation in our study was not representative of our target population, male ice hockey players who spanned the spectrum of play from youth to professional, most of whom were not elite players. In no way should frequencies of CTE reported in this sample be construed as the prevalence of CTE in the target population. Even if symptoms are not part of brain bank inclusion criteria, families whose loved ones are symptomatic former elite players are more likely to donate.^3^ The proportion of deaths due to suicide was high across the study sample, including among the lowest duration of play tertile (10 donors [38.5%]) and the non-CTE group (10 donors [28.6%]), providing further evidence for selection pressure. To evaluate these selection pressures, we used simulation to model conditions that may lead to selection bias. We considered approximately 30 000 reasonable scenarios in which duration played, CTE neuropathology, and their cross-product may be associated with selection. The median selection-adjusted OR for associations between duration played and CTE was similar to the OR found without adjusting for selection. Even under conditions of extreme selection into the brain bank, the OR had consistent magnitudes and was never null. This suggests that selection into our study was unlikely to bias outcome magnitudes, and findings regarding duration played may be generalizable to the target population.

We report duration played thresholds of 7.5 and 18 years that most closely correspond to negative and positive LRs of 0.1 and 10.2, respectively, for classifying CTE diagnosis. A diagnostic test with these LRs may produce sizable and often conclusive shifts from pretest to posttest probability.^33^ Although LRs that correspond to the thresholds were large, thresholds did not classify CTE diagnosis perfectly. For instance, there was 1 donor with CTE who played fewer than 7.5 years, and 2 donors without CTE who played more than 18 years. We also report a threshold of 16 years that maximizes sensitivity and specificity together. Expectedly, these thresholds were higher than those we previously reported for American football.^3^ These findings may inform exposure thresholds for ice hockey in future traumatic encephalopathy syndrome criteria for the clinical diagnosis of CTE but are imperfect classifiers and should not be considered thresholds below which CTE will not occur or above which CTE is guaranteed to occur. Further, thresholds were not prespecified or replicated in an independent dataset (as none exist, to our knowledge), so models may suffer from overfitting data, and threshold estimation should be interpreted with caution.

Due to the limited sample size, this study focused on ice hockey exposure as a risk factor associated with CTE-related pathology but did not investigate associations between ice hockey exposure and clinical outcomes. Clinical outcomes are more distal than direct measures of pathology and may also be due to a variety of non–CTE-related etiologies. Nonetheless, we did find an association between cumulative ptau burden (a measure of CTE severity) and 2 clinical outcomes (dementia and FAQ score), suggesting that neuropathological outcomes are clinically meaningful. Furthermore, in a much larger sample of individuals with CTE (including several of these hockey players, as well as those with other RHI exposures), we showed strong clinicopathological associations.^19^

### Strengths and Limitations

Strengths of this study include analysis of the largest cohort of ice hockey players whose brains were donated for research. To our knowledge, this is the first study to quantify ice hockey exposure as a risk factor associated with CTE neuropathological outcomes with appropriate statistical power.

There are multiple limitations to this study. First, while our study was sufficiently powered to conduct our analyses, a larger sample with a more representative distribution of hockey players across all levels played would likely provide additional insight. For example, we did not use CTE stage as an outcome because there were only approximately 10 donors in each stage, although we have shown strong association between cumulative ptau and CTE stage.^34^ Second, there is the potential for recall bias when ascertaining clinical and exposure data from informants. Some informants may not have been present for a donor’s athletic career, while the time from hockey play and informant interview differed based on age at death, and informants were aware of clinical symptoms when making statements about exposure. For professional players, online databases were used in verifying position and duration played; however, for lower-level players these data are generally unavailable online. Third, we followed similar logic to prior research using duration played in years as a proxy measure for exposure,^3^ but duration played is not an exact proxy for ice hockey–related RHI exposure. Players may have started checking at different ages, and there is considerable heterogeneity in contact across countries, levels, positions, and individual players. Additionally, at the national level, the age of the introduction of checking has changed over time. Helmet or mouthguard accelerometer data, which can more precisely track RHI severity and frequency, may prove to be beneficial in estimating risk from ice hockey play, as has been previously done for American football.^35,36,37,38^ Fourth, the study sample consisted exclusively of White males. While 95% of NHL players self-reported as White as recently as 2020, the difference between previously described football-playing donors and our hockey donors with respect to race is notable.^2,3^ Furthermore, men’s and women’s ice hockey differs by body checking rules, limiting direct comparisons. We have efforts underway to increase brain donation among female hockey players, although current numbers remain small. Fifth, some individuals who were CTE negative in this study may have developed CTE had they lived longer. Sixth, other unmeasured confounders that were upstream of and associated with both duration of hockey play and CTE pathology were not considered in our models. In general, players, parents, and doctors should carefully consider whether study findings are applicable to the setting to which they may be attempting to apply them.

## Conclusions

In this cross-sectional study of former ice hockey players, a dose-response association was observed between years of ice hockey play and CTE presence and severity. The magnitudes of outcomes in these associations remained largely unchanged after accounting for brain bank selection. CTE pathological burden was associated with dementia and impairment in instrumental activities of daily living. Similar to findings in American football, these findings implicate ice hockey play as a risk factor associated with CTE and associated clinical outcomes.

## Notes

Supplement 1.
eMethods.
**eTable 1.** Missingness of semiquantitative ptau burden data across brain regions and concussion count **eTable 2.** Sample demographic, RHI exposure, clinical, and neuropathological characteristics stratified by CTE diagnosis **eTable 3.** Sensitivity analyses assessing the association between duration played and CTE neuropathology **eFigure 1.** Selection flowchart **eFigure 2.** Receiver operating characteristic curve using duration of ice hockey play to classify CTE diagnosis
eReferences.

Supplement 2.
Data Sharing Statement
