# Supplementary material for: Duration of Ice Hockey Play and Chronic Traumatic Encephalopathy
Source: JAMA Netw Open. 2024 Dec 4;7(12):e2449106. doi: 10.1001/jamanetworkopen.2024.49106 (PMC11618473; doi:10.1001/jamanetworkopen.2024.49106)
Supplement: Supplement 1. — eMethods. eTable 1. Missingness of semiquantitative ptau burden data across brain regions and concussion count eTable 2. Sample demographic, RHI exposure, clinical, and neuropathological characteristics stratified by CTE diagnosis eTable 3. Sensitivity analyses assessing the association between duration played and CTE neuropathology eFigure 1. Selection flowchart eFigure 2. Receiver operating characteristic curve using duration of ice hockey play to classify CTE diagnosis eReferences. [file jamanetwopen-e2449106-s001.pdf]

Supplemental Online Content

Abdolmohammadi B, Tuz-Zahra F, Uretsky M, et al. Duration of ice hockey play and chronic traumatic encephalopathy. *JAMA Netw Open*. 2024;7(12):e2449106. doi:10.1001/jamanetworkopen.2024.49106

eMethods.
eTable 1. Missingness of semiquantitative ptau burden data across brain regions and concussion count
eTable 2. Sample demographic, RHI exposure, clinical, and neuropathological characteristics stratified by CTE diagnosis
eTable 3. Sensitivity analyses assessing the association between duration played and CTE neuropathology
eFigure 1. Selection flowchart
eFigure 2. Receiver operating characteristic curve using duration of ice hockey play to classify CTE diagnosis
eReferences.

This supplemental material has been provided by the authors to give readers additional information about their work.

© 2024 Abdolmohammadi B et al. *JAMA Network Open*.

eMethods

Donor Recruitment

All study brain donors belonged to the Understanding Neurological Injury and Traumatic Encephalopathy (UNITE) or Framingham Heart Study (FHS) Brain Banks. Criteria for inclusion into both studies have been previously described[1–4]. For the UNITE Brain Bank, recruitment began in 2008 and donors must have had a history of RHI exposure, from contact sport participation, military service, and/or physical violence. The FHS was established in 1948, enrolling a representative group from Framingham, Massachusetts and subsequently their children (Gen 2) and grandchildren (Gen 3), as well as ethnically diverse cohorts (Omni 1&2) to better capture changing demographics of Framingham. The FHS Brain Bank is a voluntary subgroup, with enrollment starting in 1997[4]. For both brain banks, prospective donors were excluded if there was a postmortem interval for donation greater than 72 hours, or if fragments and/or less than a hemisphere of tissue were received. In the current study, our target population was male athletes whose primary sport was ice hockey with the highest level of play spanning youth to professional play. We included donors through January 2023 from both brain banks whose primary contact sport exposure (see definition in next section) came from organized ice hockey play. We included donors from both studies to increase the sample size and improve representation across the spectrum of duration of ice hockey play (**eFigure S1**). 33 donors who played ice hockey were excluded because their primary contact sport was not ice-hockey. The next of kin or legally authorized representative of each brain donor provided written informed consent for donation around the time of death. The institutional review board at Boston University's Medical Campus (BUMC) and the VA Bedford Healthcare System approved all relevant research activities. This report followed the Strengthening the Reporting of Observational Studies in Epidemiology (STROBE) guideline for cross-sectional studies.

Contact Sport and TBI History

Retrospective data collection from informants was similar for both brain banks. Informants were aware of and reported on (see Clinical Evaluation) clinical symptoms. For each contact sport exposure (hockey or otherwise), informants provided the ages the donor began and stopped playing, the levels the donor played (youth, high school, college, juniors/semi-professional, professional), the position(s) and total years the donor played at each level. Because donors stopped playing and died at different ages, the length of time between play and informant report differed by donor. For donors who played multiple sports, we used highest level of play to define primary sport exposure. If a donor played two or more sports at the same highest level, we used the sport they played longest to define primary sport exposure. Although only donors whose primary sport was hockey were included, playing additional contact sports beyond hockey was incorporated into the statistical analyses (below).

We defined duration of ice hockey play as the total number of years of organized ice hockey play, and age of first exposure as the age the donor started playing ice hockey[5–8]. We excluded years of play in informal "men's leagues," which are difficult to quantify in terms of RHI exposure and usually do not allow checking. However, we included youth play, as checking may occur (albeit not in all leagues and we did not confirm donor-specific checking during youth play). Positions were organized into three categories: forwards (wingers and centers), defensemen, and goaltenders. For any donors who played multiple positions at their highest level of play, the position played most was noted as the primary position of play. For donors who played hockey at the professional level, position and years played were verified using a public database[9]. For former National Hockey League players, penalty minutes and fight history were collected using public databases[9,10]. In instances where data were publicly available online, online data took precedence over data collected from informants. Informants were asked whether each donor was referred to as an "enforcer" during their playing career. Informants were also asked if donors had any military service or combat exposure. Each donors' traumatic brain injury (TBI) history was queried from informants, including assessing the total number of concussions experienced after being read a formal definition of concussion[11–13].

Clinical Evaluation

For the UNITE brain bank, previously detailed methods for retrospective clinical data collection and

© 2024 Abdolmohammadi B et al. *JAMA Network Open*.

comprehensive review of all relevant medical records were followed for all participants[3,14]. A structured

© 2024 Abdolmohammadi B et al. *JAMA Network Open*.

clinical history was obtained from informants, including a timeline of cognitive, behavioral, mood and motor symptomatology. Instrumental activities of daily living were assessed using the Functional Assessment Questionnaire (FAQ)[15], completed by informants. Clinicians with expertise in neurodegenerative disease reviewed all cases to reach consensus on a dementia diagnosis based on modified Diagnostic and Statistical Manual of Mental Disorders, Fourth Edition, Text Revision (DSM-IV) criteria. Clinicians and clinical research assistants were blinded to the neuropathological examination and findings.

For the FHS brain bank, clinical data were obtained prospectively as part of the FHS clinical assessment. Participants suspected of having cognitive impairment were brought to a consensus meeting, during which it was determined whether the participant met criteria for dementia based on DSM-IV criteria as previously described[16].

Pathological Evaluation

Neuropathological evaluation for both brain banks followed similar, previously established protocols and was carried out by the same neuropathologists (A.C.M., T.D.S., B.H., V.A.). Neuropathological evaluation was performed blinded to and concurrent with clinical protocols. Tissue staining included Luxol fast blue, hematoxylin and eosin (H&E), Bielschowsky silver, ptau, α-synuclein, amyloid-β, and phosphorylated transactive response DNA binding protein 43 kDa (pTDP-43). Established criteria were used for diagnosing Alzheimer's disease (AD), Lewy body disease (LBD), Frontotemporal lobar degeneration (FTLD-TDP43, FTLD-tau, including progressive supranuclear palsy, corticobasal degeneration, Pick's, argyrophilic grain disease and tau pathology not otherwise specified) and motor neuron disease (MND). White matter rarefaction, arteriolosclerosis and atherosclerosis were assessed semi-quantitatively (0-3 scale), and micro and macro-infarcts were dichotomized as either present or absent[3,17–22].

Neuropathological diagnosis of CTE was based on the NINDS-NIBIB criteria,[23] wherein the unique pathognomonic lesion is defined as an irregular pattern of ptau aggregates in neurons and neuronal processes, with or without astrocytes, around small blood vessels and at the depths of the cortical sulci. The severity of CTE pathology was classified into four distinct stages using the McKee staging scheme that has been found to correspond to regional quantitative measures of ptau pathology[14,23,24]. In addition, neuropathologists assigned measures of semi-quantitative ptau burden across 11 regions implicated in CTE (dorsolateral middle frontal cortex, inferior orbital frontal cortex, superior temporal cortex, inferior parietal cortex, CA1 of hippocampus, CA2 of hippocampus, CA4 of hippocampus, entorhinal cortex, amygdala, substantia nigra, and locus coeruleus) on a 0-3 scale of severity.

Imputation of missing semi-quantitative ptau burden and concussion count

Missing semi-quantitative ptau values across the 11 brain regions and concussion count were imputed using multiple imputation by chained equations (MICE), creating 10 imputed datasets. Imputation models used ordinal logistic regression for ordered categorical variables and predictive mean matching for continuous variables. Imputation was informed by age at death, duration of ice hockey play, other contact sport play, position of hockey play (offense vs. other), age of first exposure to hockey play, non-missing concussion count and non-missing semi-quantitative ptau measures. Cumulative ptau burden was calculated by summing the semi-quantitative ptau measures (including both imputed and directly measured) across the 11 brain regions. Analyses below combined regression coefficients for each of the 10 datasets using Rubin's rule. **eTable S1** details missingness of semi-quantitative ptau burden data across the 11 regions and concussion count.

Statistical Analyses

We tested the association of duration of ice hockey play with two main neuropathological outcomes pertaining to CTE: CTE diagnosis and cumulative ptau burden. We utilized binary logistic regression to estimate the association of CTE diagnosis and duration of play, and linear regression to estimate the association of cumulative ptau burden and duration of play. All primary models testing associations between CTE diagnosis and cumulative ptau burden included as covariates age at death, concussion count, dichotomized participation in other contact sport play, position of hockey play (offense vs. other), and age of first exposure to hockey play as they have been hypothesized to be associated with CTE previously[1,2,8,14].

© 2024 Abdolmohammadi B et al. *JAMA Network Open*.

We evaluated the linearity assumption in several ways. For CTE diagnosis, (1) the Hosmer–Lemeshow $\chi 2$ test was used to assess goodness of fit and (2) we inspected for linearity a bar chart showing 2 + log-odds of CTE diagnosis in 3-year intervals of duration of play. For ptau burden, we inspected for linearity a locally estimated scatterplot smoothing (LOESS) regression curve with duration of hockey played on the x-axis and ptau burden on the y-axis. In preliminary analyses, we included all donors who played ice hockey, regardless of primary sport. However, in these models, linearity assumptions failed, likely from low-level players having extensive exposure to other contact sports, and we ultimately excluded donors whose primary sport was not hockey.

In 5 separate sensitivity analyses, we repeated the above analyses: (1) Adding enforcer status as a covariate to investigate if it is associated with CTE pathology independent of duration of play, (2) limiting to donors from the UNITE Brain Bank only, (3) limiting to donors with fewer than 5 years of American football play, a level of duration suggested to be predictive of CTE diagnosis[25], (4) adding an additional covariate for presence of a diagnosed tauopathy (AD, FTLD-tau) other than/in addition to CTE to help establish if other tauopathies may be contributing to the observed association, and (5) removing age at death as a covariate, as it is downstream from duration of play, and is not a confounder, but may be a potential mediator in the duration of play-CTE relationship.

We plotted a receiver operating characteristic (ROC) curve to observe how well our model classified CTE diagnosis. We identified the thresholds of duration of hockey played that corresponded to negative and positive likelihood ratios [LRs] closest to 0.1 and 10, respectively, values that may produce sizable and often conclusive shifts from pre- to post-test probability. We also identified a threshold that maximizes the sensitivity and specificity of duration of hockey played together using 3 approaches: the Youden index, the distance to the top left corner, and the concordance probability.

To quantify the conditions under which selection bias could invalidate our findings, we conducted simulation analyses using methods previously described[2]. We focused on the relationships of duration of hockey played and CTE with selection. We assumed the probability of selection, $P_i(S)$, is a function of duration played, D, CTE diagnosis, C, and their cross product, DC, in a logistic regression model, and that K is the log odds of selection when D, C, and DC are not associated with selection: $\log P_i(S)/1\text{-}P_i(S) = \beta_D D + \beta_C C + \beta_{DC} DC + K$. We set K=−1.17, the log odds of selection into a brain bank from a community-based study[26]. For each individual, i, we calculated $P_i(S)$ for a range of values of β_D, β_C, and β_{DC} (0 to 2 in intervals of 0.1) and used $1/P_i(S)$ as a weight in inverse probability weighting (IPW) analyses evaluating the association of duration played with CTE diagnosis. We limited β_D, β_C, and β_{DC} to non-negative values based on the assumption that greater duration played and the presence of CTE pathology may increase, but would not decrease probability of brain donation. For each set of values of β_D, β_C, and β_{DC}, we estimated the effect of duration played on CTE diagnosis. We repeated this approach for cumulative ptau burden.

We conducted additional analyses to provide objective evidence that players who were subjectively called enforcers were more aggressive and physical than their non-enforcer counterparts. Among NHL players (the only players with data on penalty minutes and fights), we tested the association between penalty minutes per game and fights per game and enforcer status, using a 2-sample t-test.

We tested the association of cumulative ptau burden with dementia diagnosis and FAQ scores to verify that the neuropathological changes of CTE were clinically meaningful in ice hockey players. We used binary logistic and linear regression for models with dementia and FAQ scores as outcomes respectively. Models were adjusted for age at death, duration of ice hockey play, enforcer status and dichotomized participation in other contact sport play.

All analyses were performed using SPSS Statistics version 28 and RStudio version 2023.06 .0+421. All data were collected and stored using REDCap version 13.8.1.

© 2024 Abdolmohammadi B et al. *JAMA Network Open*.

eTable S1. Missingness of semi-quantitative tau burden data across brain regions and concussion count

Region	Frequency (%) missing among brain donors before imputation (n=77)
Dorsolateral Frontal (semi-quantitative)	4 (5%)
Inferior Orbital Frontal (semi-quantitative)	12 (16%)
Superior Temporal (semi-quantitative)	2 (3%)
Inferior Parietal (semi-quantitative)	2 (3%)
CA1 hippocampal subfield (semi-quantitative)	3 (4%)
CA2 hippocampal subfield (semi-quantitative)	8 (10%)
CA4 hippocampal subfield (semi-quantitative)	12 (16%)
Entorhinal Cortex (semi-quantitative)	2 (3%)
Amygdala (semi-quantitative)	4 (5%)
Substantia Nigra (semi-quantitative)	2 (3%)
Locus Coeruleus (semi-quantitative)	15 (20%)
Concussion count (continuous)	10 (13%)

© 2024 Abdolmohammadi B et al. *JAMA Network Open*.

eTable S2. Sample demographic, RHI exposure, clinical and neuropathological characteristics, stratified by CTE diagnosis

Characteristic	No CTE No. (%) (n=35)	CTE No. (%) (n=42)	All participants No. (%) (n=77)
**Demographics**			
**Sex**			
Male	35 (100)	42 (100)	77 (100)
**Race**			
White	35 (100)	42 (100)	77 (100)
Median age at death (IQR)	48 (29-68)	52.5 (38-74)	51 (33-73)
Range	13-91	20-89	13-91
**Cause of death**			
Neurodegenerative	8 (23)	11 (26)	19 (25)
Cardiovascular disease	3 (9)	5 (12)	8 (10)
Suicide	10 (29)	12 (29)	22 (29)
Cancer	3 (9)	3 (7)	6 (8)
Motor neuron disease	0	2 (5)	2 (3)
Accidental overdose	3 (9)	4 (10)	7 (9)
Other[a]	6 (17)	4 (10)	10 (13)
Unknown	2 (6)	1 (2)	3 (4)
**Clinical Outcomes**			
Dementia	11 (31)	17 (41)	28 (36)
Mean FAQ score (SD)	6 (9.8)	11.5 (12)	9.3 (11.4)
**Repetitive Head Impact Exposure**			
Median concussion count (IQR) (n=69)	10 (0-34)	28 (14-100)	20 (10-50)
Median AFE to ice hockey (IQR)	7 (5-12)	6 (5-10)	7 (5-10)
Mean duration of play in years (SD)	11.3 (4.9)	23.3 (8.5)	17.7 (9.2)
**Highest level of play**			
Youth	2 (6)	1 (2)	3 (4)
High school	17 (49)	1 (2)	18 (23)
College	8 (23)	7 (17)	15 (20)
Juniors/Semi-Professional	7 (20)	6 (14)	13 (17)
Professional	1 (3)	27 (64)	28 (36)
**Primary position at highest level of play**			
Forward	18 (51)	27 (64)	45 (58)
Defenseman	9 (26)	14 (33)	23 (30)
Goaltender	1 (3)	0	1 (1)
Unknown	7 (20)	1 (2)	8 (10)
Enforcers	4 (11)	18 (43)	22 (29)
Military veterans	12 (34)	5 (12)	17 (22)
Other contact sport play	12 (34)	16 (38)	28 (36)
**Neuropathology**			
Mean brain weight, grams (SD)	1387.3 (146.6)	1394.9 (183.51)	1391.3 (167.1)
**CTE**			
Stage I	-	11 (26)	11 (14)
Stage II	-	11 (26)	11 (14)
Stage III	-	13 (31)	13 (17)
Stage IV	-	7 (17)	7 (9)
**Neurodegenerative diagnoses**			
Alzheimer's disease[b]	4 (11)	8 (19)	12 (16)
Lewy body disease	4 (12)	10 (24)	14 (18)
Motor neuron disease	0	2 (5)	2 (3)
Frontotemporal lobar degeneration (FTLD-tau)	1 (3)	1 (2)	2 (3)
FTLD-TDP43	0	1 (2)	1 (1)

© 2024 Abdolmohammadi B et al. *JAMA Network Open*.

No additional neurodegenerative pathology[c]	26 (74)	28 (67)	54 (70)

© 2024 Abdolmohammadi B et al. *JAMA Network Open*.

**Neuritic plaques, CERAD score** (n=75)			
None (C0)	27 (77)	27 (68)	54 (72)
Sparse (C1)	5 (14)	8 (20)	13 (18)
Moderate (C2)	3 (9)	4 (10)	7 (9)
Frequent (C3)	0	1 (3)	1 (1)
**Diffuse plaques, CERAD score** (n=75)			
None	25 (71)	23 (58)	48 (64)
Sparse	2 (6)	6 (15)	8 (11)
Moderate	2 (6)	6 (15)	8 (11)
Frequent	6 (17)	5 (13)	11 (15)
**Thal Phase** (n=66)			
Phase 0 (A0)	22 (84)	23 (58)	45 (68)
Phase 1/2 (A1)	2 (8)	3 (8)	5 (8)
Phase 3 (A2)	0	6 (15)	6 (9)
Phase 4/5 (A3)	2 (8)	8 (20)	10 (15)
**Braak Stage** (n=75)			
Stage 0 (B0)	22 (63)	14 (35)	36 (48)
Stage I/II (B1)	7 (20)	5 (13)	12 (16)
Stage III/IV (B2)	2 (6)	11 (28)	13 (17)
Stage V/VI (B3)	4 (12)	9 (23)	13 (17)
White matter rarefaction, moderate-severe (n=74)	9 (26)	13 (33)	22 (30)
Atherosclerosis, moderate-severe (n=75)	7 (20)	6 (15)	13 (17)
Arteriolosclerosis, moderate-severe (n=75)	9 (26)	11 (28)	20 (27)
TDP-43 inclusions (n=67)	0	12 (29)	12 (16)
Hippocampal sclerosis (n=67)	0	4 (10)	4 (5)
**Mean semi-quantitative ratings of ptau severity, 0-3 scale**			
Dorsolateral frontal cortex	0.39 (0.83)	1.87 (1.01)	1.19 (1.18)
Inferior orbital frontal cortex	0.21 (0.72)	1.52 (1.01)	1.03 (1.11)
Superior temporal cortex	0.53 (1.08)	1.48 (1.11)	1.04 (1.19)
Inferior parietal cortex	0.41 (0.89)	1.25 (1.24)	0.86 (1.16)
CA1	0.7 (1.19)	1.43 (1.22)	1.1 (1.25)
CA2	0.34 (0.77)	1.1 (1.12)	0.78 (1.05)
CA4	0.13 (0.45)	1.2 (1.14)	0.8 (1.07)
Entorhinal	0.79 (1.1)	1.62 (1.15)	1.24 (1.19)
Amygdala	0.71 (1.09)	1.55 (1.18)	1.15 (1.21)
Substantia nigra	0.6 (1.52)	0.95 (1.01)	0.79 (1.28)
Locus coeruleus	0.63 (0.82)	1.89 (0.97)	1.39 (1.1)

[a]Other causes of death included infection (n=2), motor vehicle accidents (n=2), alcoholic cirrhosis, Covid-19, stroke, acute necrotizing pancreatitis, and other unspecified causes (n=2).

[b]NIA-Reagan Intermediate or high

[c]Donors without CTE had no neurodegenerative pathology, while donors with CTE had no neurodegenerative pathology in addition to CTE

Abbreviations: AFE, age of first exposure; CERAD, Consortium to Establish a Registry for Alzheimer's Disease; CTE, chronic traumatic encephalopathy; FAQ, functional activities questionnaire; IQR, interquartile range; SD, standard deviation.

Sample sizes for neuropathological outcomes ranged from 66 to 77, as certain measures were not assessed for all donors.

© 2024 Abdolmohammadi B et al. *JAMA Network Open*.

eTable S3. Sensitivity analyses assessing the relationship between duration played and CTE neuropathology

A) Relationship between duration played and CTE neuropathology in participants from the UNITE Brain Bank	
**Outcome**	**Association magnitude (95% confidence interval)**
1) *CTE diagnosis (n=42 with CTE, 26 without CTE)*<br>Duration, per year	*Odds ratio of having CTE*<br>1.31 (1.11; 1.54); p=0.001
2) *Cumulative ptau burden*<br>Duration, per year	*Standardized unit increase in PTAU burden*<br>0.035 (0.011; 0.058); p=0.005

B) Relationship between duration played and CTE neuropathology in participants with <5 years of American football play	
**Outcome**	**Association magnitude (95% confidence interval)**
1) *CTE diagnosis (n=35 with CTE, 32 without CTE)*<br>Duration, per year	*Odds ratio of having CTE*<br>1.43 (1.18; 1.74); p<0.001
2) *Cumulative ptau burden*<br>Duration, per year	*Standardized unit increase in PTAU burden*<br>0.035 (0.015; 0.056); p<0.001

C) Relationship between duration played and CTE neuropathology, adjusting for presence of non-CTE tauopathies, including AD and FTLD-tau	
**Outcome**	**Association magnitude (95% confidence interval)**
1) *CTE diagnosis*<br>Duration, per year	*Odds ratio of having CTE*<br>1.35 (1.16-1.56); p<0.001
2) Cumulative ptau burden<br>Duration, per year	*Standardized unit increase in cumulative ptau burden*<br>0.035 (0.016-0.053); p<0.001

D) Relationship between duration played and CTE neuropathology, excluding age at death	
**Outcome**	**Association magnitude (95% confidence interval)**
3) *CTE diagnosis*<br>Duration, per year	*Odds ratio of having CTE*<br>1.35 (1.17-1.57); p<0.001
4) Cumulative ptau burden<br>Duration, per year	*Standardized unit increase in cumulative ptau burden*<br>0.054 (0.03-0.077); p<0.001

All models were adjusted for age at death, concussion count, dichotomized participation in other contact sport play, position of hockey play (offense vs. other), and age of first exposure to hockey play (except for panel D, in which the model did not adjust for age at death)
Abbreviation: AD: Alzheimer's disease, FTLD-tau: frontotemporal lobar degeneration-tau.

© 2024 Abdolmohammadi B et al. *JAMA Network Open*.

eFigure S1. Selection Flowchart

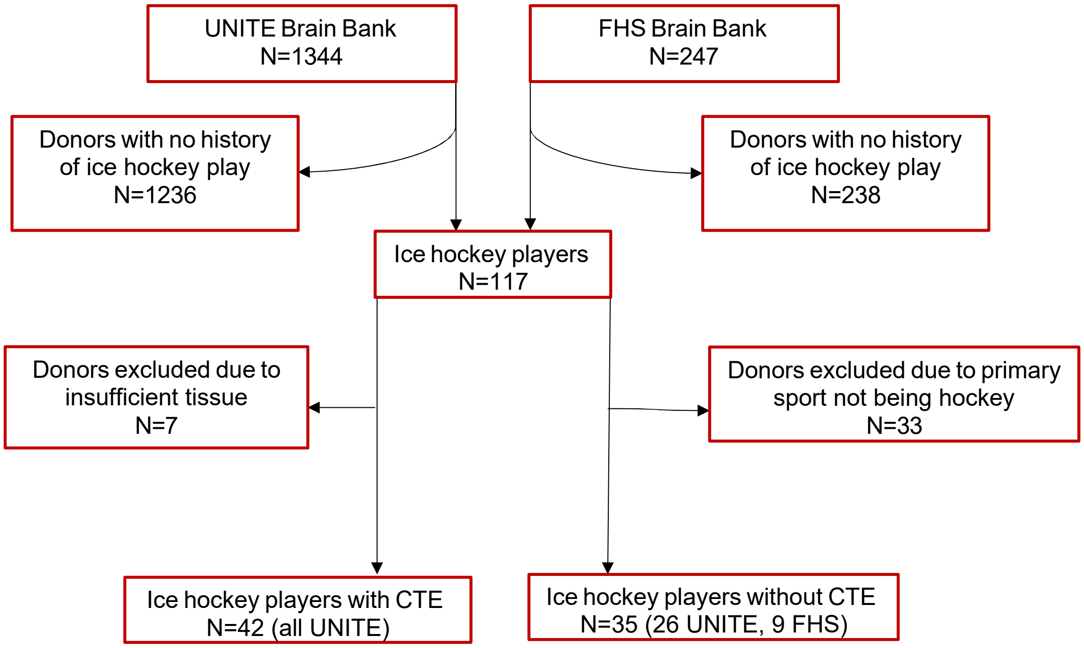

© 2024 Abdolmohammadi B et al. *JAMA Network Open*.

eFigure S2. Receiver operating characteristic curve utilizing duration of ice hockey play to classify CTE diagnosis

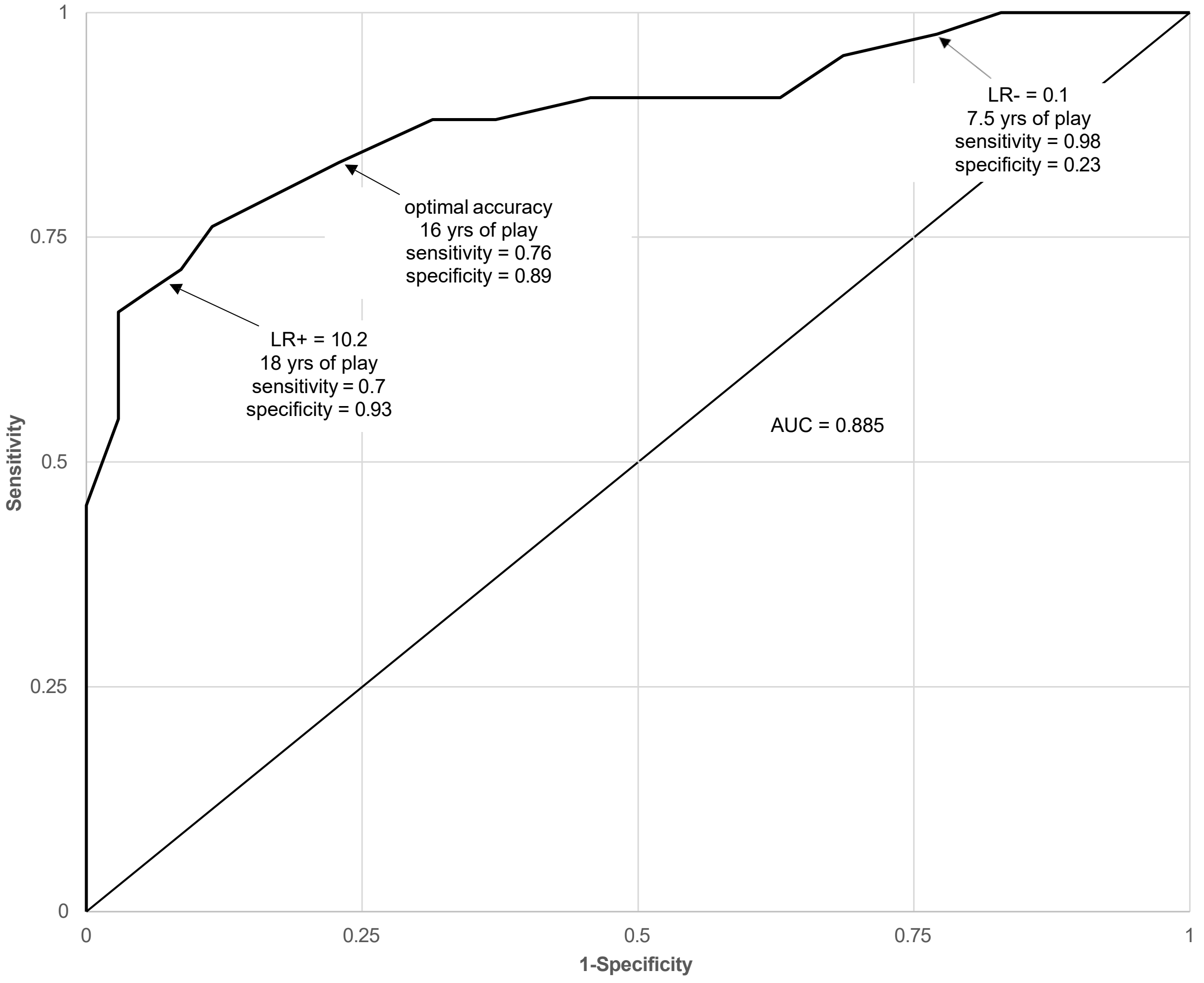

CTE classification sensitivity and specificity were maximized at 16 years of play. Thresholds of duration of play corresponding to negative and positive likelihood ratios (LR-, LR+) of 0.1 and 10.2 were 7.5 years and 18 years, respectively. Abbreviation: AUC, area under the curve. CTE, chronic traumatic encephalopathy.

© 2024 Abdolmohammadi B et al. *JAMA Network Open*.

References

1. Mez J, Daneshvar DH, Kiernan PT, et al. Clinicopathological Evaluation of Chronic Traumatic Encephalopathy in Players of American Football. *JAMA*. 2017;318(4):360-370. doi:10.1001/jama.2017.8334

2. Mez J, Daneshvar DH, Abdolmohammadi B, et al. Duration of American Football Play and Chronic Traumatic Encephalopathy. *Ann Neurol*. 2020;87(1):116-131. doi:10.1002/ana.25611

3. Mez J, Solomon TM, Daneshvar DH, et al. Assessing clinicopathological correlation in chronic traumatic encephalopathy: rationale and methods for the UNITE study. *Alzheimers Res Ther*. 2015;7(1):62. doi:10.1186/s13195-015-0148-8

4. Au R, Seshadri S, Knox K, et al. The Framingham Brain Donation Program: neuropathology along the cognitive continuum. *Curr Alzheimer Res*. 2012;9(6):673-686. doi:10.2174/156720512801322609

5. Guskiewicz KM, McCrea M, Marshall SW, et al. Cumulative effects associated with recurrent concussion in collegiate football players: the NCAA Concussion Study. *JAMA*. 2003;290(19):2549-2555. doi:10.1001/jama.290.19.2549

6. Stamm JM, Koerte IK, Muehlmann M, et al. Age at First Exposure to Football Is Associated with Altered Corpus Callosum White Matter Microstructure in Former Professional Football Players. *J Neurotrauma*. 2015;32(22):1768-1776. doi:10.1089/neu.2014.3822

7. Stamm JM, Bourlas AP, Baugh CM, et al. Age of first exposure to football and later-life cognitive impairment in former NFL players. *Neurology*. 2015;84(11):1114-1120. doi:10.1212/WNL.0000000000001358

8. Alosco ML, Mez J, Tripodis Y, et al. Age of First Exposure to Tackle Football and Chronic Traumatic Encephalopathy. *Ann Neurol*. 2018;83(5):886-901. doi:10.1002/ana.25245

9. Hockey Reference. Professional Hockey Database. Accessed July 14, 2022. https://www.hockey-reference.com/

10. HockeyFights. HockeyFights. Accessed February 16, 2024. https://www.hockeyfights.com/

11. Corrigan JD, Bogner J. Initial reliability and validity of the Ohio State University TBI Identification Method. *J Head Trauma Rehabil*. 2007;22(6):318-329. doi:10.1097/01.HTR.0000300227.67748.77

12. Seichepine DR, Stamm JM, Daneshvar DH, et al. Profile of self-reported problems with executive functioning in college and professional football players. *J Neurotrauma*. 2013;30(14):1299-1304. doi:10.1089/neu.2012.2690

13. Robbins CA, Daneshvar DH, Picano JD, et al. Self-reported concussion history: impact of providing a definition of concussion. *Open Access J Sports Med*. 2014;5:99-103. doi:10.2147/OAJSM.S58005

14. McKee AC, Stern RA, Nowinski CJ, et al. The spectrum of disease in chronic traumatic encephalopathy. *Brain*. 2013;136(Pt 1):43-64. doi:10.1093/brain/aws307

15. Pfeffer RI, Kurosaki TT, Harrah CHJ, Chance JM, Filos S. Measurement of functional activities in older adults in the community. *J Gerontol*. 1982;37(3):323-329. doi:10.1093/geronj/37.3.323

16. Chêne G, Beiser A, Au R, et al. Gender and incidence of dementia in the Framingham Heart Study from mid-adult life. *Alzheimers Dement*. 2015;11(3):310-320. doi:10.1016/j.jalz.2013.10.005

© 2024 Abdolmohammadi B et al. *JAMA Network Open*.

17. McKeith IG. Consensus guidelines for the clinical and pathologic diagnosis of dementia with Lewy bodies (DLB): report of the Consortium on DLB International Workshop. *J Alzheimers Dis*. 2006;9(3 Suppl):417-423. doi:10.3233/jad-2006-9s347

18. Vonsattel JPG, Del Amaya MP, Keller CE. Twenty-first century brain banking. Processing brains for research: the Columbia University methods. *Acta Neuropathol*. 2008;115(5):509-532. doi:10.1007/s00401-007-0311-9

19. Beekly DL, Ramos EM, van Belle G, et al. The National Alzheimer's Coordinating Center (NACC) Database: an Alzheimer disease database. *Alzheimer Dis Assoc Disord*. 2004;18(4):270-277.

20. Mackenzie IRA, Neumann M, Bigio EH, et al. Nomenclature for neuropathologic subtypes of frontotemporal lobar degeneration: consensus recommendations. *Acta Neuropathol*. 2009;117(1):15-18. doi:10.1007/s00401-008-0460-5

21. Brownell B, Oppenheimer DR, Hughes JT. The central nervous system in motor neurone disease. *J Neurol Neurosurg Psychiatry*. 1970;33(3):338-357. doi:10.1136/jnnp.33.3.338

22. Consensus recommendations for the postmortem diagnosis of Alzheimer's disease. The National Institute on Aging, and Reagan Institute Working Group on Diagnostic Criteria for the Neuropathological Assessment of Alzheimer's Disease. *Neurobiol Aging*. 1997;18(4 Suppl):S1-2.

23. Bieniek KF, Cairns NJ, Crary JF, et al. The Second NINDS/NIBIB Consensus Meeting to Define Neuropathological Criteria for the Diagnosis of Chronic Traumatic Encephalopathy. *J Neuropathol Exp Neurol*. Published online February 21, 2021. doi:10.1093/jnen/nlab001

24. Alosco ML, Cherry JD, Huber BR, et al. Characterizing tau deposition in chronic traumatic encephalopathy (CTE): utility of the McKee CTE staging scheme. *Acta Neuropathol*. 2020;140(4):495-512. doi:10.1007/s00401-020-02197-9

25. Katz DI, Bernick C, Dodick DW, et al. National Institute of Neurological Disorders and Stroke Consensus Diagnostic Criteria for Traumatic Encephalopathy Syndrome. *Neurology*. 2021;96(18):848-863. doi:10.1212/WNL.0000000000011850

26. Haneuse S, Schildcrout J, Crane P, Sonnen J, Breitner J, Larson E. Adjustment for selection bias in observational studies with application to the analysis of autopsy data. *Neuroepidemiology*. 2009;32(3):229-239. doi:10.1159/000197389

© 2024 Abdolmohammadi B et al. *JAMA Network Open*.
