# Supplementary material for: Duration of Ice Hockey Play and Chronic Traumatic Encephalopathy
Source: JAMA Netw Open. 2024 Dec 4;7(12):e2449106. doi: 10.1001/jamanetworkopen.2024.49106 (PMC11618473; doi:10.1001/jamanetworkopen.2024.49106)
Supplement: Supplement 2. — Data Sharing Statement [file jamanetwopen-e2449106-s002.pdf]

Data Sharing Statement

Abdolmohammadi. Duration of Ice Hockey Play and Chronic Traumatic Encephalopathy. *JAMA Netw Open*. Published online December 4, 2024. doi:10.1001/jamanetworkopen.2024.49106

Data
Data available: Yes
Data types: Deidentified participant data, Data dictionary
How to access data: Data can be accessed on FITBIR: https://fitbir.nih.gov. In addition, requests can be made via the BU ADRC: https://wwwru.bumc.bu.edu/BUADC/RequestClinicalData.aspx
When available: With publication

Supporting Documents
Document types: None

Additional Information
Who can access the data: Data will be made available to approved principal investigators.
Types of analyses: Analyses should have deliverables such as manuscripts, grants and/or training.
Mechanisms of data availability: For access directly from BU ADRC, a proposal should be submitted through the website and approved by study leaders. A data sharing fee may be assessed to cover support costs.
